# Root attributes dominate the community assembly of soil fungal functional guilds across arid inland river basin

**DOI:** 10.3389/fmicb.2022.938574

**Published:** 2022-07-22

**Authors:** Yin Wang, Jianming Wang, Mengjun Qu, Jingwen Li

**Affiliations:** ^1^School of Ecology and Nature Conservation, Beijing Forestry University, Beijing, China; ^2^Ejina Institute of Populus euphratica, Beijing Forestry University, Beijing, China

**Keywords:** root attributes, community assembly, soil fungi, fungal functional guilds, arid inland river basins

## Abstract

Plant attributes are increasingly acknowledged as key drivers shaping soil fungal communities, but considerable uncertainty exists over fungal community assembly mechanisms and their plant drivers based only on inferences from plant aboveground attributes. To date, empirical evidences of how root attributes are integrated into microbiome–plant linkages remain limited. Using 162 soil samples from a typical arid inland river basin in China, we assessed the drivers that regulate the distribution patterns and assembly processes of total, mycorrhizal, saprotrophic and pathotrophic fungi in surface (0–15 cm) and subsurface soils (15–30 cm). Total fungi and fungal functional guilds exhibited similar distribution patterns in arid inland river basins. Null-model and variance partitioning analysis revealed that the heterogeneous selection induced by root attributes, rather than dispersal limitation, predominated the fungal community assembly. Multiple regressions on matrices further demonstrated that specific root length were the most important predictors of fungal community assembly, which mediated the balance of assembly processes of soil fungal communities. Heterogeneous selection decreased for total, mycorrhizal and saprotrophic fungi, but increased for pathotrophic fungi with increasing specific root length. Additionally, fine-root biomass exerted important effects on fungal assembly processes in subsurface soil but not in surface soil, suggesting root attributes differently affected fungal community assembly between surface and subsurface soil. Collectively, our study highlights the importance of considering root attributes in differentiating the balance of stochastic and deterministic processes in microbial community assembly.

## Introduction

Elucidating the fundamental assembly process underpinning the distribution patterns of soil microorganisms is key to understanding the maintenance mechanism of belowground biodiversity in natural ecosystems (Stegen et al., 2013; Jiao and Lu, 2020). Community assembly, a major hotspot of ecological research, is widely known to be explained by two most broadly embraced theories: niche and neutral theory (Stegen et al., 2013; Meyer et al., 2018; Ning et al., 2020). The former emphasizes that deterministic processes largely modulate community assembly, and community composition can be convergent due to a similar environment (homogeneous selection), while divergence in community composition may arise from heterogeneous environments (heterogeneous selection) (Stegen et al., 2012). Conversely, the latter considers that individuals are ecologically equivalent in communities, and community assembly is regulated by stochastic process (including dispersal limitation and ecological drift, Chase and Myers, 2011; Zhou and Ning, 2017). After long-standing debates, it is widely acknowledged that both stochastic and deterministic processes jointly govern soil microbial assembly (Hubbell, 2001; Vellend, 2010; Ning et al., 2020). However, it is still challenging to characterize the relative contribution of deterministic and stochastic processes on soil microbial assembly, due to their relative roles depend on ecosystem types and environmental conditions (Dini-Andreote et al., 2015).

Soil microorganisms, especially fungi, exhibit a high level of biodiversity on the Earth and have successfully colonized all natural ecosystems (Nilsson et al., 2019). It is widely believed that soil fungi play key roles in nutrient cycling, organic matter decomposition and plant health (Delgado-Baquerizo et al., 2016; Li et al., 2019; Chen et al., 2020). These ecological functions are implemented by distinct functional guilds: mycorrhizal, saprotrophic and pathotrophic fungi (Nguyen et al., 2016). Mycorrhizal fungi can form symbiotic associations with 86% of all plant species, facilitating nutrient uptake of plants (van der Heijden et al., 2015). Saprotrophic fungi are the primary decomposers of dead plant biomass (Bardgett and van der Putten, 2014), while pathotrophic fungi can cause disease, affecting the survival of their hosts (García-Guzmán and Heil, 2014). Owing to the difference in lifestyle, dispersal ability, and nutrient acquisition strategies, different soil fungal functional guilds may exhibit divergent distribution patterns (Zheng et al., 2021). However, the difference in assembly processes among soil fungal functional guilds remains relatively poorly understood. Hence, taking fungal functional guilds into account might enrich our understanding of fungal assembly mechanisms.

Fungal community assembly may be affected by a variety of biotic factors, such as plant diversity, traits, and biomass (Delgado-Baquerizo et al., 2018; Prada-Salcedo et al., 2021). Plant traits and biomass largely determine the quantity and quality of plant litter or root exudates, which are believed to play crucial roles in affecting fungal community assembly (Pei et al., 2016; Leff et al., 2018). While plant attributes appear to greatly contribute to fungal community assembly, abiotic factors (i.e., spatial distance and edaphic factors) are also likely to modify the effects of plant attributions on soil fungi (Guo et al., 2020; Zhang et al., 2020). Indeed, plant communities in arid regions are characterized by high root: shoot ratios, high species turnover rate and even divergent functional traits (Ottaviani et al., 2020; Wang et al., 2020), shaped by the prevailing extreme environmental conditions, such as hyperaridity, paucity of soil nutrients, and high variability of water availability. Moreover, the influence of soil factors and water availability on plant communities may differ between above- and belowground dimension (Carvajal et al., 2019). Hence, plant above- and belowground attributes may play different roles in affecting soil fungal community assembly. However, the relative roles of abiotic and biotic factors (especially plant aboveground and root attributes) in shaping fungal community assembly remain poorly understood.

To elucidate assembly processes of total, mycorrhizal, saprotrophic, and pathotrophic fungi and their potential drivers in surface and subsurface soils, this study investigated soil fungal community composition, plant community composition, leaf and root attributes and edaphic properties at 27 sites across a typical arid inland river basin. Here, we hypothesized that (1) heterogeneous selection induced by plant attributes shapes the distribution patterns of soil fungal functional guilds; (2) root attributes are dominant drivers in shaping fungal community assembly; (3) root attributes mediate the effect of heterogeneous selection on fungal community assembly, while their roles differ between surface and subsurface soil.

## Materials and methods

### Study region and sampling design

**FIGURE 1:**
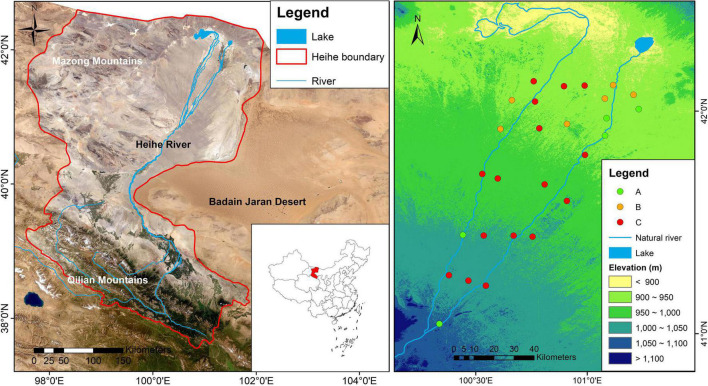
Geographic locations of the Heihe river basin and its main topographic features and 27 sampling sites. The datasets originate from National Tibetan Plateau Data Center http://data.tpdc.ac.cn, and then the map was performed with ArcGIS 10. **(A)** Temperate broadleaf deciduous forest (*Populus euphratica* Woodland); **(B)** Temperate broadleaf deciduous scrub (*Tamarix ramosissima* scrub); **(C)** desert vegetation.

A field survey was carried out in the lower reaches of the Heihe river, Inner Mongolia, Northwest China (Figure 1), where elevation ranges from 903 to 1064 m, and mean annual potential evapotranspiration surpasses 3,390 mm (Ding et al., 2017). Narrow ranges of annual mean precipitation (29–42 mm) and annual mean temperature (8.50–8.94°C) have been observed in these regions from 1960 to 2020, indicating limited climatic changes in the study area. The soils are predominantly Gypsi Sali-Orthic Aridosols (Gong, 2014; correspond to Gypsisols, IUSS Working Group WRB), which typically contain more sand particles (Supplementary Table 1). The study regions cover mainly three vegetation types. The desert riparian forests which are dominated by Temperate broadleaf deciduous forest (*Populus euphratica* Woodland) and Temperate broadleaf deciduous scrub (*Tamarix ramosissima* scrub) mainly occur near the river bed with a shallower water table and higher soil moisture. The desert vegetation which is dominated by drought-tolerant desert species, such as *Reaumuria soongarica* and *Ephedra przewalskii* is mainly distributed in desert region with a deeper water table and far from river channels (Supplementary Table 2).

Field sampling was conducted in early July, 2020, and 27 sites were selected at different distances from the riverbed (Figure 1). At each site without anthropogenic or natural disturbance, three 20 m by 20 m quadrats were established randomly within a 1 km by 1km quadrat under representative landscape and dominant vegetation (a total of 81 quadrats). Plant species abundance and composition were recorded at the quadrat level. Each site had a monitoring well equipped with a Diver water level logger (Eijkelkamp, Netherlands) to record groundwater data (every 30 min). Groundwater data were obtained from 27 wells in 2020. Among these wells, 18 wells were established in 2017 and 9 wells were established in 2010 (Zhu et al., 2014). The distance from the Heihe river represents a gradient of natural groundwater depth, because groundwater is mainly recharged by the intermittent river (Wang et al., 2019). Seasonal variations and ranges of groundwater depth are provided (Supplementary Table 3 and Supplementary Figure 1). Based on groundwater data, we calculated groundwater depth seasonality (standard deviation of monthly groundwater depth, SDGWD) and mean groundwater depth (MGWD, m).

### Sampling and measurement of soil

To reduce the effects of soil heterogeneity, 20–30 soil cores were randomly collected within 20m by 20m quadrats (under the canopy of plants) from two soil layers (0–15 cm and 15–30 cm). After roots and visible organic debris were removed, soil cores from two soil layers were separately composited. 162 soil samples at two depths were passed through a 2 mm mesh sieve and divided into three subsamples: two subsamples were stored at –20°C for molecular analysis and measurement of soil available nutrients, and the other was stored at 4°C for soil physiochemical analyzes.

In the laboratory, soil moisture (SM, %) was measured gravimetrically by drying at 105°C to constant weight. Soil electrical conductivity (indicator of soil soluble salt, SEC, ms/cm) and soil pH were measured at a soil-to-water ratio of 1:5 and 1:2.5. Soil organic carbon (SOC, g/kg) was measured by the K_2_Cr_2_O_7_ oxidation method (Garland et al., 2021). Soil total nitrogen (STN, g/kg) was measured by Kjeldahl procedure. Soil ammonium nitrogen (NH_4_N, mg/kg) and soil nitrate nitrogen (NO_3_N, mg/kg) were measured by the KCl extraction method (Schimel and Bennett, 2004). Soil total phosphorus (STP, g/kg) was measured by the molybdenum blue method. Soil available phosphorus (SAP, mg/kg) was measured by the Sodium bicarbonate extraction method (Vitousek, 2004).

### Sampling and measurement of plant attributes

Four leaf or root traits related to plant adaptation strategies were measured for all plant species. At least 20 fully expanded and sun-exposed leaves were sampled from 5 individuals of each species for measuring leaf traits, and all leaves from a selected species in each quadrat were regarded as a composite sample. Leaf samples were scanned on a Scanner (Epson V800) at a resolution of 4800 × 6000 dpi. ImageJ (Schindelin et al., 2015) was used to obtain leaf area (LA, cm^2^). Finally, leaf samples were dried at 60°C to record dry mass. Specific leaf area (SLA, cm^2^/g) was calculated as leaf area per unit dry mass. Leaf carbon concentration (LCC, mg/g) was measured using K_2_Cr_2_O_7_ oxidation method. Leaf phosphorus concentration (LPC, mg/g) was measured using colorimetry following H_2_O_2_-H_2_SO_4_ digestion. Leaf nitrogen concentration (LNC, mg/g) was measured using an elemental analyzer (FLASH2000, Elementar, American).

Plant fine-roots were harvested as follows. For herbaceous plants, we sampled the whole plants to separate fine-roots. For woody plants, we first removed the surrounding soil near the stem, and then traced the taproot to the main lateral roots. Until fine-roots appeared, we cut fine-root from an intact lateral root cluster. However, desert plant species often have a limited amount of fine-roots in water-limited environments. Therefore, at least 6 individuals per species were present per quadrat. In the laboratory, fine-roots were washed of soil. WinRHIZO 2009 (Regent Instruments, Canada) was applied to obtain root length (RL, m) and root diameter (RD, mm). Fine-roots were dried at 60°C to constant weight and dry mass was recorded. Specific root length (SRL, m/g) was calculated as root length per unit dry mass. Dried fine-root samples were used to measure root carbon (RCC, mg/g), phosphorus (RPC, mg/g) and nitrogen (RNC, mg/g) concentration, following the measuring methods of leaf nutrient traits.

For root biomass sampling, 30–40 soil cores (6 cm in diameter, 1 m in height) were collected randomly in each quadrat. Soil cores were sieved by a 0.5 mm mesh, and fine-roots (diameter ≤ 2 mm) were picked out. Then, fine-roots were washed of soil and dried at 60°C for measuring fine-root biomass (FRB, kg). According to a previous study in these regions, the distribution of fine-root biomass for most plant species can be found in the 0–1 m soil layer (fine-root biomass > 90%, Wang et al., 2021). As a result, measuring biomass in the 0–1 m soil layer is a representative sampling method.

Community weighted means were calculated (CWM, Garnier et al., 2004). CWM indicates the dominant trait value of species in the plant community and reflects how plants acquire resources in distinct environments (Carvajal et al., 2019). CWM was calculated as $\text{CWM}=\sum_{n=1}^{\text{S}}{\text{P}}_{n}\times {\text{Trait}}_{n}$, where P*_*n*_* was the relative abundance of species *n* in each quadrat, and Trait*_*n*_* was the mean trait value of species *n* in each quadrat.

### Molecular and bioinformatics analysis

Total DNA were extracted from fresh soil samples (500 mg) using the PowerSoil DNA Isolation Kit (MoBio Laboratories, Carlsbad, CA, United States) according to the manual. The primers ITS1F (5′-CTTGGTCATTTAGAGGAAGTAA-3′) and ITS2 (5′-TGCGTTCTTCATCGATGC-3′) were used to amplify the ITS region for fungi. PCR reactions were conducted in triplicate for each sample under the following conditions: 95°C for 5 min, 28 cycles of 95°C for 45 s, 55°C for 50 s, and 72°C for 45 s with a final extension at 72°C for 10 min. PCR amplicons were extracted from 2% agarose gels and purified using the Agencourt AMPure XP Kit (Axygen Biosciences, Tewksbury, MA, United States) following standard protocols. The purified products were pooled in equimolar amounts, and paired-end sequencing (2 × 300 bp) was performed on an Illumina MiSeq platform (Illumina, San Diego, CA, United States) at Allwegene Tech, Ltd. (Beijing, China).

The acquired sequences less than 230 bp with ambiguous base calls and low quality scores (≤ 20) were discarded using QIIME (Caporaso et al., 2010). The qualified sequences were clustered into operational taxonomic units (OTUs) according to 97% pairwise identity using UPARSE (Edgar, 2013). The BLAST method was used to identity the taxonomy of each ITS sequence *via* comparisons against sequences in the UNITE database. OTUs not identified as fungi were removed from the OTU abundance matrices. Finally, to correct the effects of distinct sequencing depths, the OTU matrices were rarefied to the lowest number of sequences (23,883 for fungi) detected within all samples. Three major functional guilds, including mycorrhizal (arbuscular mycorrhizal and ectomycorrhizal fungi), saprotrophic and pathotrophic fungi were determined according to the criteria of Nguyen et al. (2016). Only highly probable and probable guilds with an identified single trophic mode were included in this study. Given the low occurrence and ambiguous life style of endophytes, they were not included in our analysis.

## Data analysis

Considering that not all plant attributes and abiotic factors have normal frequency distributions, SM, SEC, SAP, NH_4_N, and NO_3_N were square root transformed, and LA, SLA, RL, SRL, and RNC were log-10 transformed before analysis.

The variation in species composition among plant communities was calculated as pairwise Bray-Curtis dissimilarity. Standardized Euclidean distance matrices were computed for single leaf or root attributes and soil factors. Spatial distance matrices were calculated based on GPS coordinates.

The abundance-weighted null deviation approach (Bray–Curtis dissimilarity) was applied to clarify the assembly processes of total fungi and fungal functional guilds. Here, the regional species abundance distributions were fixed, and null communities were generated by randomly resampling individuals to a local community (Zhang et al., 2020; Liu et al., 2021). *β*-deviations (the standardized effect size of *β*-diversity) were calculated as the difference of the observed *β* diversity from the mean *β* diversity of 999 null communities. If communities are dominated by stochastic processes, *β*-deviations would be statistically indistinguishable from 0. Moreover, *β*-deviations higher than 0 imply a dominant effect of dispersal limitation or heterogeneous selection, while *β*-deviations lower than 0 indicate a dominant effect of homogeneous selection or homogenizing dispersal (Zhang et al., 2020).

In order to test the impact of abiotic and biotic factors on the assembly processes of soil fungi functional guilds, Mantel tests were performed to evaluate the relationship of abiotic and biotic factors with *β*-deviations. Multiple regressions on matrices (MRM) approach was used to explore the major abiotic and biotic drivers of fungal community assembly. To avoid data overfitting, all factors were subjected to forward-selection until *P* < 0.05. Then, variation partitioning analysis (VPA) was conducted to further examine the relative contribution of environmental selection and dispersal limitation. Pure effects of biotic and abiotic factors (except space factors) represent the influences of environmental selection, while pure effects of space factors represent the influences of dispersal limitation (Zhang et al., 2020). Fungi community differences were visualized using non-metric multidimensional scaling analysis (NMDS) based on Bray–Curtis dissimilarity. The significance of community differences among three vegetation types was checked using permutational analysis of variance (PERMANOVA).

Partial Mantel tests were performed to evaluate the relationships between *β*- deviations and selected drivers after accounting for the influence of other abiotic and biotic factors. All samples were further divided into different categories to test the variations in assembly processes along the gradient of selected abiotic and biotic drivers.

## Results

Approximately 6,248,043 and 5,939,706 sequences were obtained from soil fungi in surface and subsurface soils. The ITS data set was subsampled to 23,883 reads for soil fungi per sample. Sequences from surface and subsurface soils were clustered into 6,530 and 5,836 OTUs, respectively. Among them, 4,125 and 3,591 OUTs were identified as three functional guilds, which occupied more than 50% of the total sequences (Supplementary Figure 2). Saprotrophic fungi were the dominant functional guilds in surface and subsurface soils (1,269 and 1,100 OTUs), followed by mycorrhizal fungi (676 and 520 OTUs) and pathotrophic fungi (306 and 272 OTUs). Three major functional guilds accounted for approximately 52.5% and 56.6% of the total functional sequences in surface and subsurface soils, respectively (Supplementary Table 4).

### Community assembly processes of total, mycorrhizal, saprotrophic, and pathotrophic fungi

Non-metric multidimensional scaling analysis (NMDS) showed that community dissimilarities (Bray–Curtis dissimilarity) of total, mycorrhizal, saprotrophic and pathotrophic fungi differed significantly among three vegetation types (PERMANOVA, *P* < 0.001; Supplementary Figure 3). Community dissimilarities of all functional guilds exhibited gradual shifts along the vegetation gradient (from desert riparian forests to desert).

**FIGURE 2:**
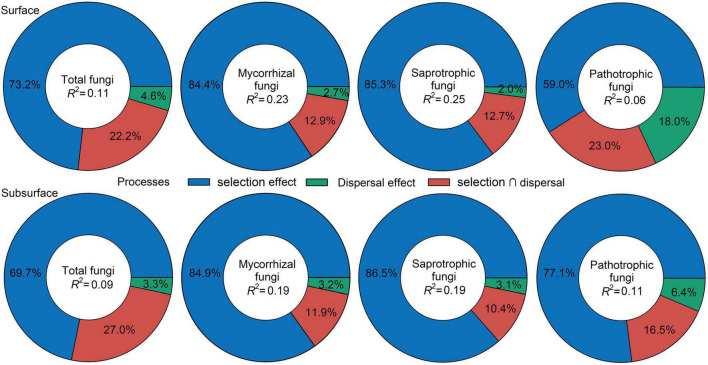
Summary of the relative effects of environmental selection, dispersal limitation and their interaction derived from the variation partitioning analysis.

Null-model analysis revealed that *β*-deviations of total, mycorrhizal, saprotrophic and pathotrophic fungi in surface and subsurface soils were consistently higher than zero (Table 1), suggesting a dominant role of dispersal limitation or heterogeneous selection in shaping fungal community assembly. VPA further assessed the relative contribution of dispersal limitation and environmental selection, indicating that environmental selection had greater effect on total fungi and three functional guilds in surface and subsurface soils (Figure 2).

**TABLE 1:** One-sample two-sided t-test was used to compare mean *β* deviation against zero for total, mycorrhizal, saprotrophic and pathotrophic fungi in surface and subsurface soil.

Trophic groups	Surface soil		Subsurface soil	
	Mean *β* deviation	*P*	Mean *β* deviation	*P*
Total fungi	298.54	<0.001	300.70	<0.001
Mycorrhizal fungi	37.17	<0.001	53.52	<0.001
Saprotrophic fungi	174.64	<0.001	179.53	<0.001
Pathotrophic fungi	84.29	0.001	122.86	<0.001

### The relative importance of root vs. leaf attributes and abiotic factors on fungal community assembly

**FIGURE 3:**
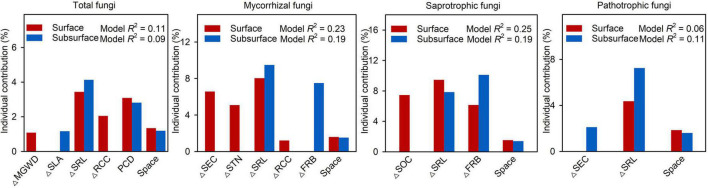
Results of the multiple regressions on distance matrices (MRM) for *β*-deviations of total, mycorrhizal, saprotrophic and pathotrophic fungi in surface and subsurface soil. PCD, plant community dissimilarity; SLA, specific leaf area; SRL, specific root length; RCC, root carbon concentration; FRB, fine-root biomass; MGWD, mean groundwater depth; SEC, soil electric conductivity; STN, soil total nitrogen; SOC, soil organic carbon.

Mantel test showed that *β*-deviations of total, mycorrhizal, saprotrophic and pathotrophic fungi were significantly related to abiotic and biotic factors in surface and subsurface soils (Supplementary Table 5). MRM revealed that abiotic and biotic factors jointly explained 11.0%, 22.5%, 24.6%, and 6.2% of the variation in total, mycorrhizal, saprotrophic and pathotrophic fungi in surface soils, while abiotic and biotic factors together explained 9.3%, 18.5%, 19.4%,and 11.0% of the variation in total, mycorrhizal, saprotrophic and pathotrophic fungi in subsurface soils, respectively (Supplementary Table 6). Among the selected predictors, root attributes (SRL and FRB) had the most important effect on *β*-deviations of total fungi and three functional guilds after controlling for the influence of other important factors (Figure 3). Among root attributes, SRL was the most important driver shaping *β*-deviations of total (3.4% and 4.1%), mycorrhizal (8.0% and 9.5%), saprotrophic (9.5% and 7.9%) and pathotrophic (4.4% and 7.3%) fungi in surface and subsurface soils. Moreover, FRB was also a crucial driver determining *β*-deviations of mycorrhizal (7.5%) and saprotrophic (10.1%) fungi in subsurface soils (Supplementary Table 6).

### Effects of root attributes on the assembly processes of fungal communities

**FIGURE 4:**
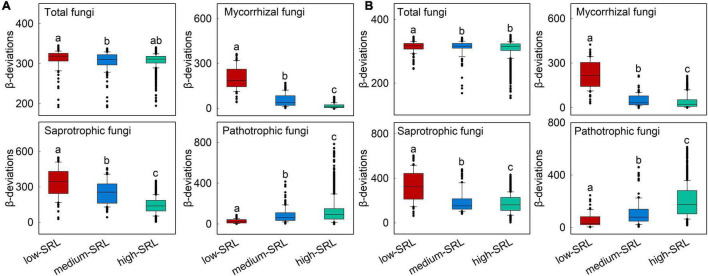
Variations in *β*-deviations of mycorrhizal, saprotrophic and pathotrophic fungi across low, medium, and high categories of specific root length (SRL) in surface **(A)** and subsurface **(B)** soil. SRL was separated into three categories and correlated with *β*-deviations within each category. Boxplots that do not share a lower case letter are significantly different (*P* < 0.05, Wilcoxon rank-sum test). These three categories were divided based on Supplementary Figure 4.

**FIGURE 5:**
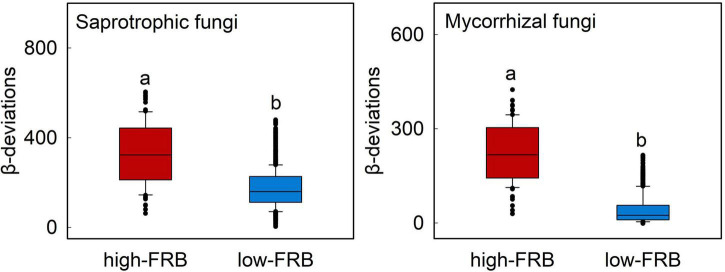
Variations in *β*-deviations of mycorrhizal and saprotrophic fungi across low and high categories of fine-root biomass (FRB) in subsurface soil. FRB was separated into two categories and correlated with *β*-deviations within each category. Boxplots that do not share a lower case letter are significantly different (*P* < 0.05, Wilcoxon rank-sum test). These two categories were divided based on Supplementary Figure 4.

The relationship between *β*-deviations of total, mycorrhizal, saprotrophic and pathotrophic fungi and major root attributes was used to evaluate the variation in relative importance of deterministic and stochastic processes. Partial Mantel tests also indicated that root attributes were significantly correlated with *β*-deviations after controlling for the influence of other key factors (Table 2), as evinced by the pairwise comparisons of *β*-deviations with different categories of SRL or FRB (Figure 4 and Supplementary Figure 4). *β*-deviations significantly decreased with increasing SRL, suggesting that increasing SRL reduced the relative influence of heterogeneous selection on total, mycorrhizal and saprotrophic fungi in surface and subsurface soils but promoted the relative influence of heterogeneous selection on pathotrophic fungi (Figure 4). In subsurface soils, *β*-deviations of mycorrhizal and saprotrophic fungi were significantly higher in high-FRB sites (Figure 5), indicating a dominant effect of heterogeneous selection on mycorrhizal and saprotrophic fungi in high-FRB sites.

**TABLE 2:** Relationships between root attributes and *β*-deviations of total, mycorrhizal and saprotrophic and pathotrophic fungi in surface and subsurface soil.

Surface soil	Total fungi	Mycorrhizal fungi	Saprotrophic fungi	Pathotrophic fungi
PCD	0.136***			
SRL	0.176***	0.284***	0.271***	–0.125*
RCC	0.040*	–0.070		
FRB			0.194***	
PCD	0.109**			
SRL	0.160***	0.255***	0.186**	–0.189***
FRB		0.199**	0.257**	

Partial Mantel tests are applied to investigate these relationships when controlling for the influence of other biotic and abiotic factors. PCD, plant community dissimilarity; SRL, specific root length; RCC, root carbon concentration; FRB, fine-root biomass; SLA, specific leaf area. *, ** and *** significant at probability level of 0.05, 0.01 and 0.001, respectively.

## Discussion

### Heterogeneous selection regulated fungal community assembly across arid inland river basin

Disentangling the assembly mechanisms controlling fungal distribution patterns is a research hotspot in ecology (Zhou and Ning, 2017; Meyer et al., 2018; Jiao and Lu, 2020). The difference in distribution patterns and assembly processes among total fungi and three ecologically important fungal functional guilds: mycorrhizal, saprotrophic and pathotrophic fungi, has received little attention so far in arid inland river basins, a superior habitat for belowground biota in arid regions. Here, similar shifts for total fungi and three functional guilds along the vegetation gradient were observed in surface and subsurface soils (Supplementary Figure 3), implying that total, mycorrhizal, saprotrophic and pathotrophic fungi exhibited similar distribution patterns. MRM further demonstrated that both abiotic and biotic factors were responsible for the *β*-deviations of total, mycorrhizal, saprotrophic and pathotrophic fungi (Figure 2), suggesting that deterministic and stochastic processes jointly shaped soil fungal community assembly. According to VPA results, the relative contribution of environmental selection on soil fungal community assembly was higher than that of dispersal limitation (Figure 2). Null-model results also revealed a dominant effect of dispersal limitation or heterogeneous selection on fungal community assembly (Table 1). These results collectively implied that the community assembly of total, mycorrhizal, saprotrophic and pathotrophic fungi depended largely on heterogeneous selection, which is inconsistent with the findings in agricultural ecosystems that homogeneous selection has a dominant effect on fungal community assembly (Jiao and Lu, 2020), probably because environmental heterogeneity (e.g., wide soil moisture range 0.1–17%, obvious vegetation changes, Supplementary Table 3) was greater in natural ecosystems than in agricultural ecosystems. Moreover, it is also noteworthy that a large proportion of the variation in fungal *β*-deviations remained unexplained by selected abiotic and biotic factors (Supplementary Table 6). This result was consistent with the findings of previous studies, which may reflect the effects of other unidentified factors (e.g., plant litter or exudates properties; Yang et al., 2019; Guo et al., 2020). Together, heterogeneous selection played a greater role than stochastic processes in shaping the community assembly of total, mycorrhizal, saprotrophic and pathotrophic fungi.

### Root attributes as the most important drivers of the assembly processes of different fungal functional guilds

In the present study, we observed that the *β*-deviations of total, mycorrhizal, saprotrophic and pathotrophic fungi could be partially explained by abiotic and biotic factors, but biotic and abiotic factors (except space factor) had a stronger influence than spatial distance (Figure 2). Traditional studies have emphasized that plant aboveground attributes are the predominant drivers of fungal community assembly (Leff et al., 2018; Wang et al., 2020; Liu et al., 2021). However, our MRM results showed that root attributes exerted the most prominent effects on the *β*-deviations of total, mycorrhizal, saprotrophic and pathotrophic fungi (Figure 3 and Supplementary Table 6). Moreover, the dominant roles played by root attributes as drivers of fungal community assembly still emerged within each vegetation types (Supplementary Figures 5, 6). Altogether, our study showed that root attributes, rather than leaf attributes measured in the present study, determined community assembly of soil fungal functional guilds. These novel findings highlight that above- and belowground plant attributes should be simultaneously combined to seek for a more comprehensive understanding of the linkages between plant and fungal communities.

Among root attributes, SRL had the strongest influence on the community assembly of total, mycorrhizal, saprotrophic and pathotrophic fungi in surface and subsurface soils (Figure 3). We found that *β*-deviations of total, mycorrhizal and saprotrophic fungi were positively associated with the difference in SRL (Supplementary Table 5), indicating that the relative importance of heterogeneous selection was significantly increased by heterogeneity in biotic conditions resulted from divergence in SRL. Specifically, the relative importance of heterogeneous selection in regulating total, mycorrhizal and saprotrophic fungi were greater in low SRL habitats (group A, Temperate broadleaf deciduous forest, Figure 4). There are several possible reasons. First, it is clear that heterogeneity in biotic conditions is higher in low SRL habitats than in high SRL habitats (group C, desert vegetation, Supplementary Table 7). Such higher heterogeneity in low SRL habitats may led to high variation in fungal community structure, thereby increased strength of heterogeneous selection (Zhou and Ning, 2017). Second, decreasing RD (and therefore increasing SRL) can help plants to cope with seasonally inhospitable conditions in the desert because thin-rooted species can explore greater lengths of soil per unit of carbon invested (high SRL) with less reliance on mycorrhizal fungi (Kong et al., 2014; Ma et al., 2018), and thus decrease the power of heterogeneous selection. Furthermore, the consistent relationship between assembly processes and SRL for saprotrophic and mycorrhizal fungi may be owing to their positive biotic feedbacks. For example, the synergy between saprotrophic fungi and mycorrhizal fungi can accelerate litter decomposition in nutrient-limited environments (Frey, 2019; Cao et al., 2022). Notably, we found positive linkages between SRL and the assembly processes of pathotrophic fungi (Figures 4, 5). The most likely explanation is that the linkages can be altered by mycorrhizal fungi, as a result of the pronounced suppression effect of mycorrhiza on pathotrophic fungi. For example, mycorrhizal fungi can enhance plant resistance to pathotrophic fungi (Sikes et al., 2009; Cameron et al., 2013; Laliberté et al., 2015). Our result showed an increasing and a decreasing trend in the relative abundance of pathotrophic and mycorrhizal fungi, respectively along the SRL gradient (Supplementary Figure 7), which is similar to the findings of a previous study (Semchenko et al., 2018) that plants with higher SRL are related to more diverse pathotrophic fungi. Taken together, our study reveals that root attributes, as the most important drivers, governed the relative importance of heterogeneous selection in structuring soil fungal community.

### Root attributes exerted different effects on the assembly of fungal guilds between surface and subsurface soil

Although our results found that SRL play a key role in soil fungal assembly processes, other predictor such as fine-root biomass (FRB) similarly explained a significant fraction of variation in soil fungal functional guilds (Figure 3). For instance, FRB exerted significantly direct influence on mycorrhizal and saprotrophic fungi, and this impact mainly acted in the subsurface soil but were weaker in the surface soil (Figure 3). Meanwhile, FRB contributed to structuring the assembly processes of mycorrhizal and saprotrophic fungi in subsurface soil within three vegetation types (Supplementary Figure 6). This suggests that fungal community assembly in subsurface soil is more related to the amount of resources (i.e., fine-root biomass) than in the surface soil, which is only related to the quality of resources (i.e., root traits). The differential responses found for mycorrhizal and saprotrophic fungi in surface and subsurface soil imply that plant communities exert different effects on soil fungi in different soil layers, mediated by different root attributes. A plausible explanation of these results might be based on the differences in available substrates and host specificity. It is widely believed that the decomposition of litter often takes place in surface soils, especially in arid regions (Jobbágy and Jackson, 2001). Less accessible substrates at greater depths may select for specialized taxa adapted to resource-limited conditions (Baldrian, 2009; Whalen et al., 2021). Accordingly, FRB may represent major and available organic substrates (e.g., root litter and exudates, Eisenhauer et al., 2017; Sokol et al., 2019) for saprotrophic fungi in subsurface soils, thereby regulating community assembly of saprotrophic fungi. Moreover, mycorrhizal fungi are widely known to utilize photosynthate in fine roots and provide a more efficient alternative to roots in the acquisition of nutrients and water under harsh environment (Begum et al., 2019). Due to nutrient and water availability decreased with increasing soil depth (Supplementary Table 8), more roots may rely on mycorrhizal fungi in order to acquire limiting resource in subsurface soil, in turn affect community assembly of mycorrhizal fungi.

Our findings also highlight the potential role played by FRB as a mediator of assembly processes of mycorrhizal and saprotrophic fungi in subsurface soil. Specifically, the relative effects of heterogeneous selection on mycorrhizal and saprotrophic fungi were higher in high-FRB sites (Figure 5). The dominant effect of heterogeneous selection on mycorrhizal fungi in high-FRB sites may be attributed to the mycorrhizal status of dominant plant species. The dominant species of high-FRB sites (*Populus euphratica*) is a typical arbuscular mycorrhizal tree (Yang et al., 2013), which has strong biotic interactions with mycorrhizal fungi and thus significantly enhances the power of selection processes. Furthermore, higher FRB are concomitant with more available resources, which in turn promote the proliferation of specialized saprotrophs in subsurface soils (Chen et al., 2020), thereby mediating the effect of selection processes on saprotrophic fungi. Overall, these results further confirmed root attributes mediate the effect of heterogeneous selection on fungal community assembly, while their roles differ between surface and subsurface soil.

## Conclusion

This study conducted a systematic investigation into the distribution patterns and the assembly processes of total, mycorrhizal, saprotrophic and pathotrophic fungi in surface and subsurface soil layers across an arid inland river basin of China. Our study provides empirical evidence that community assembly of total fungi and fungal functional guilds was governed to a great extent by root attributes based heterogeneous selection, rather than by dispersal limitation. SRL mediated the balance of assembly processes of soil fungal communities, and heterogeneous selection decreased for total, mycorrhizal and saprotrophic fungi, but increased for pathotrophic fungi with increasing SRL. Moreover, FRB only explained a significant fraction of variations in mycorrhizal and saprotrophic fungi in subsurface soil, suggesting root attributes differently influence fungal community assembly between surface and subsurface soil. Our study highlights the central role of root attributes in predicting the assembly processes of fungal community assembly and suggests that combining above- and belowground plant attributes (e.g., litter attributes) will strengthen our understanding of the linkages between plant and fungal communities.

## Data availability statement

The datasets presented in this study can be found in online repositories. The names of the repository/repositories and accession number(s) can be found below: https://www.ncbi.nlm.nih.gov/, PRJNA771521.

## Author contributions

YW, JW, and JL conceived and designed the experiments. YW, JW, and MQ performed the field investigation and collected the data. YW and JW analyzed the data. YW contributed reagents, materials, and analysis tools. YW, JW, and JL wrote the manuscript. All authors contributed to the article and approved the submitted version.

## Conflict of interest

The authors declare that the research was conducted in the absence of any commercial or financial relationships that could be construed as a potential conflict of interest.

## Publisher’s note

All claims expressed in this article are solely those of the authors and do not necessarily represent those of their affiliated organizations, or those of the publisher, the editors and the reviewers. Any product that may be evaluated in this article, or claim that may be made by its manufacturer, is not guaranteed or endorsed by the publisher.
