# Supplementary material for: Root attributes dominate the community assembly of soil fungal functional guilds across arid inland river basin
Source: Front Microbiol. 2022 Jul 22;13:938574. doi: 10.3389/fmicb.2022.938574 (PMC9355615; doi:10.3389/fmicb.2022.938574)
Supplement: Supplementary file 1 [file Data_Sheet_1.docx]

# Supplementary Material

**Table S1** Soil particle composition for all sampling sites.

| Soil depth | Soil particle composition (%) | | |
| --- | --- | --- | --- |
|  | Soil sand content (> 50 µm) | Soil silt content (2–50 µm) | Soil clay content (< 2 µm). |
| 0–15cm | 57.5 a  (6.7) | 32.9 b  (5.5) | 9.5 c  (1.5) |
| 15–30cm | 61.6 a  (8.4) | 29.5 b  (6.8) | 8.9 c  (1.7) |

Soil particle composition was analyzed using a Mastersizer 2000 laser particle size analyzer (Malvern Instruments, United Kingdom) that divided the soil into sand (> 50 µm), silt (2–50 µm), and clay (< 2 µm). Values are means (standard deviation). Values at the same columns followed by different letters differed significantly at *P* < 0.05 (ANOVA, Tukey’s HSD test).

**Table S2** Information on sampling sites in our study region

| **Site** | **Mean (Range) groundwater depth (m)** | **Distance from the river channel (m)** | **Elevation (m)** | **Vegetation type** | **Soil moisture 0~15 cm (%)** | **Soil moisture 15~30 cm (%)** |
| --- | --- | --- | --- | --- | --- | --- |
| 1 | 1.30 (0.38−1.70) | 32 | 959 | A | 9.7±0.8 | 11.5±1.9 |
| 2 | 1.78 (1.43−2.16) | 47 | 1,010 | A | 8.5±0.9 | 6.5±0.3 |
| 3 | 1.80 (1.52−2.00) | 99 | 939 | A | 9.9±1.0 | 4.4±1.3 |
| 4 | 1.96 (1.76−2.23) | 104 | 915 | A | 8.9±1.8 | 8.7±7.9 |
| 5 | 2.30 (2.17−2.48) | 230 | 1,064 | A | 9.3±2.0 | 7.8±0.3 |
| 6 | 2.12 (1.77−2.38) | 643 | 907 | B | 4.8±1.9 | 4.3±1.9 |
| 7 | 2.93 (2.37−3.23) | 1,698 | 951 | B | 1.8±0.6 | 1.3±0.6 |
| 8 | 4.75 (4.39−4.97) | 2,821 | 914 | B | 1.2±1.4 | 0.8±1.3 |
| 9 | 5.04 (4.92−5.31) | 3,397 | 903 | B | 0.7±0.2 | 0.2±0.2 |
| 10 | 3.81 (3.71−3.92) | 3,249 | 945 | B | 1.7±0.6 | 1.2±0.6 |
| 11 | 2.53 (2.32−2.62) | 831 | 946 | B | 4.5±1.5 | 0.9±0.5 |
| 12 | 2.70 (2.48−2.96) | 641 | 1,027 | C | 0.6±0.4 | 0.3±0.1 |
| 13 | 3.15 (3.09−3.21) | 3,610 | 1,012 | C | 1.4±0.9 | 0.2±0.1 |
| 14 | 3.54 (3.43−3.59) | 1,941 | 945 | C | 0.9±0.6 | 0.1±0.1 |
| 15 | 3.12 (2.93−3.22) | 912 | 976 | C | 0.2±0.1 | 0.3±0.1 |
| 16 | 3.92 (3.47−4.13) | 2,562 | 1,032 | C | 0.8±0.7 | 2.6±1.4 |
| 17 | 4.21 (4.18−4.24) | 4,288 | 993 | C | 0.6±0.3 | 0.2±0.2 |
| 18 | 6.68 (6.66−6.70) | 5,745 | 976 | C | 0.3±0.0 | 0.2±0.0 |
| 19 | 6.24 (6.21−6.26) | 4,842 | 932 | C | 0.2±0.1 | 0.3±0.1 |
| 20 | 4.00 (3.78−4.18) | 4,285 | 1,027 | C | 0.5±0.1 | 1.4±2.3 |
| 21 | 5.45 (5.27−5.68) | 4,029 | 960 | C | 0.4±0.3 | 0.3±0.1 |
| 22 | 2.68 (2.48−2.85) | 1,867 | 1,009 | C | 1.0±0.2 | 1.8±2.1 |
| 23 | 2.85 (2.43−3.01) | 649 | 980 | C | 0.7±0.1 | 0.2±0.1 |
| 24 | 3.39 (2.65−3.91) | 2,098 | 926 | C | 0.4±0.2 | 0.2±0.1 |
| 25 | 3.79 (3.64−3.98) | 4,300 | 928 | C | 0.4±0.2 | 0.2±0.1 |
| 26 | 2.77 (2.61−2.91) | 2,097 | 937 | C | 1.1±0.2 | 0.7±0.6 |
| 27 | 2.78 (2.35−2.93) | 1,429 | 985 | C | 0.9±0.2 | 0.4±0.2 |

Soil moisture values are means ± standard deviation. A, Temperate broadleaf deciduous forest (*Populus euphratica* Woodland); B, Temperate broadleaf deciduous scrub (*Tamarix ramosissima* scrub); C, desert vegetation.

**Table S3** Numbers of reads and OTUs belonging to the indicated functional groups

| Layers | Functional groups | Total Reads | Mean Reads | Total OTUS | Mean OTUS |
| --- | --- | --- | --- | --- | --- |
| Surface | Total fungi | 1,934,523 | 23883 | 6,530 | 293.10 ± 15.52 |
|  | Mycorrhizal fungi | 66,705 | 823.52 ± 175.32 | 676 | 19.07 ± 2.01 |
|  | Saprotrophic fungi | 548,737 | 6774.53 ± 551.55 | 1,269 | 62.05 ± 3.23 |
|  | Pathotrophic fungi | 86,735 | 1070.80 ± 187.64 | 306 | 15.00 ± 0.85 |
| Subsurface | Total fungi | 1,934,523 | 23883 | 5,836 | 257.81 ± 11.21 |
|  | Mycorrhizal fungi | 68,450 | 845.06 ± 124.77 | 520 | 14.90 ± 1.42 |
|  | Saprotrophic fungi | 459,245 | 5669.69 ± 424.00 | 1,100 | 53.41 ± 2.46 |
|  | Pathotrophic fungi | 99,045 | 1222.78 ± 216.44 | 272 | 13.48 ± 0.66 |

Mean Reads and OTUS values are means ± standard deviation.

**Table S4** Mantel test showing relationships between biotic, abiotic factors and β-deviations of total, mycorrhizal, saprotrophic and pathotrophic fungi in surface and subsurface soil

| **Factors** | **Surface** | | | | **Subsurface** | | | |
| --- | --- | --- | --- | --- | --- | --- | --- | --- |
|  | **Total fungi** | **Mycorrhizal fungi** | **Saprotrophic fungi** | **Pathotrophic fungi** | **Total fungi** | **Mycorrhizal fungi** | **Saprotrophic fungi** | **Pathotrophic fungi** |
| Spatial distance | 0.172*** | 0.187** | 0.191** | -0.160** | 0.162*** | 0.168*** | 0.162*** | -0.158** |
| ∆MGWD | 0.149*** |  |  |  | 0.104*** |  | 0.133* | -0.140* |
| ∆SDGWD | 0.181*** | 0.326*** | 0.262* |  | 0.126*** | 0.261*** | 0.290*** | -0.211** |
| ∆pH |  |  |  |  | 0.114*** | 0.109* | 0.239** |  |
| ∆SM | 0.226*** | 0.370*** | 0.346*** | -0.199** | 0.178*** | 0.321*** | 0.244** | -0.194** |
| ∆SEC | 0.177*** | 0.340*** | 0.294** | -0.149* |  |  |  | -0.140* |
| ∆SOC | 0.181*** | 0.308*** | 0.408*** | -0.228** | 0.158*** | 0.280*** | 0.222** | -0.228** |
| ∆STN | 0.108*** | 0.299*** | 0.211** |  | 0.152*** | 0.197* | 0.210* | -0.245** |
| ∆NH_4_N |  | 0.362*** | 0.322** |  | 0.103** | 0.127* | 0.173* |  |
| ∆NO_3_N |  |  |  |  |  |  |  |  |
| ∆STP | 0.109*** | 0.189** |  |  |  |  |  |  |
| ∆SAP |  |  |  |  |  |  |  |  |
| ∆Sand | 0.116*** | 0.213** | 0.344*** | -0.130* | 0.171*** | 0.260*** | 0.209** | -0.239** |
| ∆Slit | 0.116*** | 0.229** | 0.351*** | -0.137* | 0.164*** | 0.261** | 0.206** | -0.243** |
| ∆Clay | 0.099*** | 0.121* | 0.229*** |  | 0.184*** | 0.238*** | 0.206* | -0.225** |
| PCD | 0.257*** | 0.119** | 0.213*** |  | 0.229*** | 0.116** | 0.140** |  |
| ∆SLA | 0.117*** | 0.196** |  |  | 0.151*** |  |  |  |
| ∆LCC |  |  |  |  |  |  |  |  |
| ∆LNC |  |  |  |  |  |  |  |  |
| ∆LPC |  |  |  |  |  |  |  |  |
| ∆RD | 0.154*** | 0.192** | 0.160** | -0.116* | 0.109*** | 0.138* | 0.276*** | -0.205*** |
| ∆SRL | 0.265*** | 0.369*** | 0.439*** | -0.225*** | 0.249*** | 0.383*** | 0.363*** | -0.280*** |
| ∆RCC | 0.231*** | 0.158*** | 0.261*** | -0.168*** | 0.173*** | 0.219*** | 0.180** | -0.086* |
| ∆RNC | 0.188*** | 0.130** | 0.246*** | -0.157*** | 0.226*** | 0.281*** | 0.307*** | -0.161** |
| ∆RPC | 0.214*** | 0.254*** | 0.338*** | -0.193*** | 0.196*** | 0.197*** | 0.230*** | -0.115* |
| ∆FRB | 0.108** | 0.258*** | 0.367*** | -0.200** | 0.120** | 0.348*** | 0.387*** | -0.214*** |

MGWD, mean groundwater depth; SDGWD, groundwater depth seasonality; SM, soil moisture; SEC, soil electric conductivity; SOC, soil organic carbon; STN, soil total nitrogen; NH_4_N, soil ammonium nitrogen; NO_3_N, soil nitrate nitrogen; STP, soil total phosphorus, SAP, soil available phosphorus; Sand, Soil sand content (> 50 µm); Slit, Soil silt content (2–50 µm); Clay, Soil clay content (< 2 µm); PCD, plant community dissimilarity; SLA, Specific leaf area; LCC, Leaf carbon concentration; LNC, Leaf nitrogen concentration; LPC, Leaf phosphorus concentration; RD, root diameter; SRL, Specific root length; RCC, Root carbon concentration; RNC, Root nitrogen concentration; RPC, Root phosphorus concentration; FRB, fine-root biomass.

**Table S5** Results of the multiple regressions on distance matrices (MRM) for β-deviations of total, mycorrhizal, saprotrophic and pathotrophic fungi in surface and subsurface soil. See abbreviations in Table S4.

| **Soil layer** | **Trophic groups** | **Variable retained in the model and its individual contribution (%)** | **Model *R*^2^** | **Model *P*** |
| --- | --- | --- | --- | --- |
| Surface | Total fungi | ∆SRL (3.4), PCD (3.1), ∆RCC (2.0), ∆MGWD (1.1), Spatial distance (1.4). | 0.11 | <0.0001 |
|  | Mycorrhizal fungi | ∆SRL (8.0), ∆SEC (6.6), ∆STN (5.1), Spatial distance (1.6), ∆RCC (1.2). | 0.23 | <0.0001 |
|  | Saprotrophic fungi | ∆SRL (9.5), ∆SOC (7.5), RBD (6.1), Spatial distance (1.5). | 0.25 | <0.0001 |
|  | Pathotrophic fungi | ∆SRL (4.4), Spatial distance (1.8). | 0.06 | <0.0001 |
| Subsurface | Total fungi | ∆SRL (4.1), PCD (2.8), ∆SLA (1.2), Spatial distance (1.2). | 0.09 | <0.0001 |
|  | Mycorrhizal fungi | ∆SRL (9.5), RBD (7.5), Spatial distance (1.5). | 0.19 | <0.0001 |
|  | Saprotrophic fungi | RBD (10.1), ∆SRL (7.9), Spatial distance (1.4). | 0.19 | <0.0001 |
|  | Pathotrophic fungi | ∆SRL (7.3), ∆SEC (2.1), Spatial distance (1.6). | 0.11 | <0.0001 |

**Table S6** Heterogeneity in biotic conditions for each group. Heterogeneity was estimated by computing the average dissimilarity between sites (Huber et al., 2020).

| Group | A | B | C |
| --- | --- | --- | --- |
| Heterogeneity in biotic conditions (specific root length) | 0.410 | 0.349 | 0.306 |

A, Temperate broadleaf deciduous forest (*Populus euphratica* Woodland); B, Temperate broadleaf deciduous scrub (*Tamarix ramosissima* scrub); C, desert vegetation.

We computed a Euclidean distance matrix and calculated the dissimilarity between sites (Ed) as follows:

$$Ed=\frac{Euc}{Euc_{max}}+0.001$$

where Euc is the Euclidean distance between two sites and Euc_max_ corresponds to the maximum Euclidean distance considering all the pairwise distances in the overall dataset. 0.001 was added to account for zero similarity between sites. Then, we calculated the mean Ed of each computed similarity matrix and used it as an index of environmental heterogeneity in each group.

Huber, P., Metz, S., Unrein, F. et al. Environmental heterogeneity determines the ecological processes that govern bacterial metacommunity assembly in a floodplain river system. ISME J 14, 2951–2966 (2020).

**Table S7** Plant and soil attributes among three vegetation types.

| Vegetation type | Plant abundance | SLA | LCC | LNC | LPC | RCC |
| --- | --- | --- | --- | --- | --- | --- |
| A | 108.40 a  (39.15) | 61.05 a  (5.50) | 351.19 a  (6.06) | 19.32 a  (2.13) | 1.25 a  (0.06) | 562.25 a  (2.35) |
| B | 108.78 a  (31.22) | 44.53 b  (4.24) | 347.50 a  (19.80) | 17.11 b  (0.91) | 1.33 a  (0.06) | 511.60 b  (8.32) |
| C | 78.00 b  (10.03) | 35.50 c  (1.75) | 435.29 b  (7.93) | 15.15 c  (0.45) | 1.31 a  (0.03) | 483.48 c  (3.21) |
| Vegetation type | RNC | RPC | MGWD | SDGWD | SM  (0~15cm) | SM  (15~30cm) |
| A | 6.46 a  (0.31) | 0.82 a  (0.03) | 1.83 a  (0.09) | 0.25 a  (0.02) | 9.26 a  (0.33) | 6.40 a  (1.13) |
| B | 10.65 b  (0.54) | 1.35 b  (0.06) | 3.53 b  (0.27) | 0.13 b  (0.01) | 2.46 b  (0.45) | 1.45 b  (0.38) |
| C | 13.48 c  (0.39) | 1.54 c  (0.03) | 3.83 b  (0.18) | 0.09 c  (0.01) | 0.65 c  (0.06) | 0.60 b  (0.15) |

Values at the same columns followed by different letters differed significantly at *P* < 0.05 (ANOVA, Tukey’s HSD test). A, Temperate broadleaf deciduous forest (*Populus euphratica* Woodland); B, Temperate broadleaf deciduous scrub (*Tamarix ramosissima* scrub); C, desert vegetation. See abbreviations in Table S4.


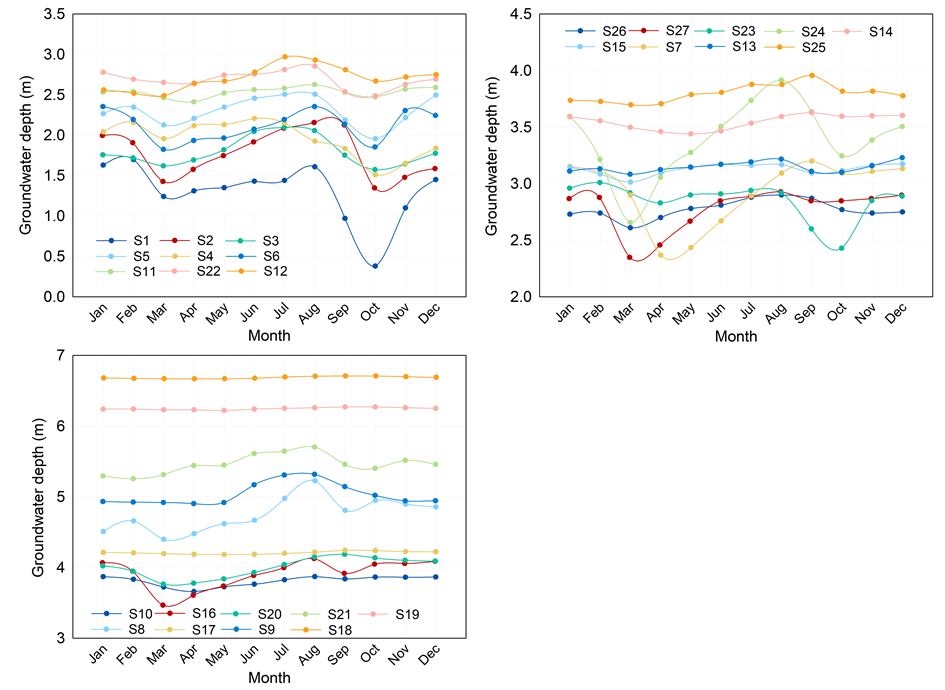


**Figure S1** Groundwater depth of each study site (from January 2020 to December 2020)


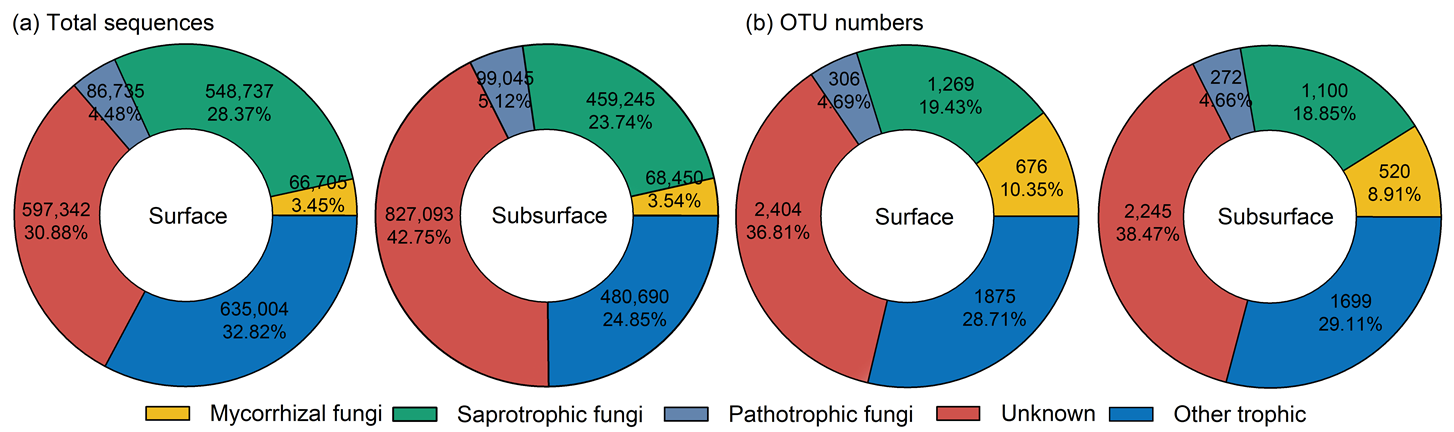


**Figure S2** Sequences (a) and operational taxonomic unit numbers (b) of total, mycorrhizal, saprotrophic and pathotrophic fungi in surface and subsurface soil.


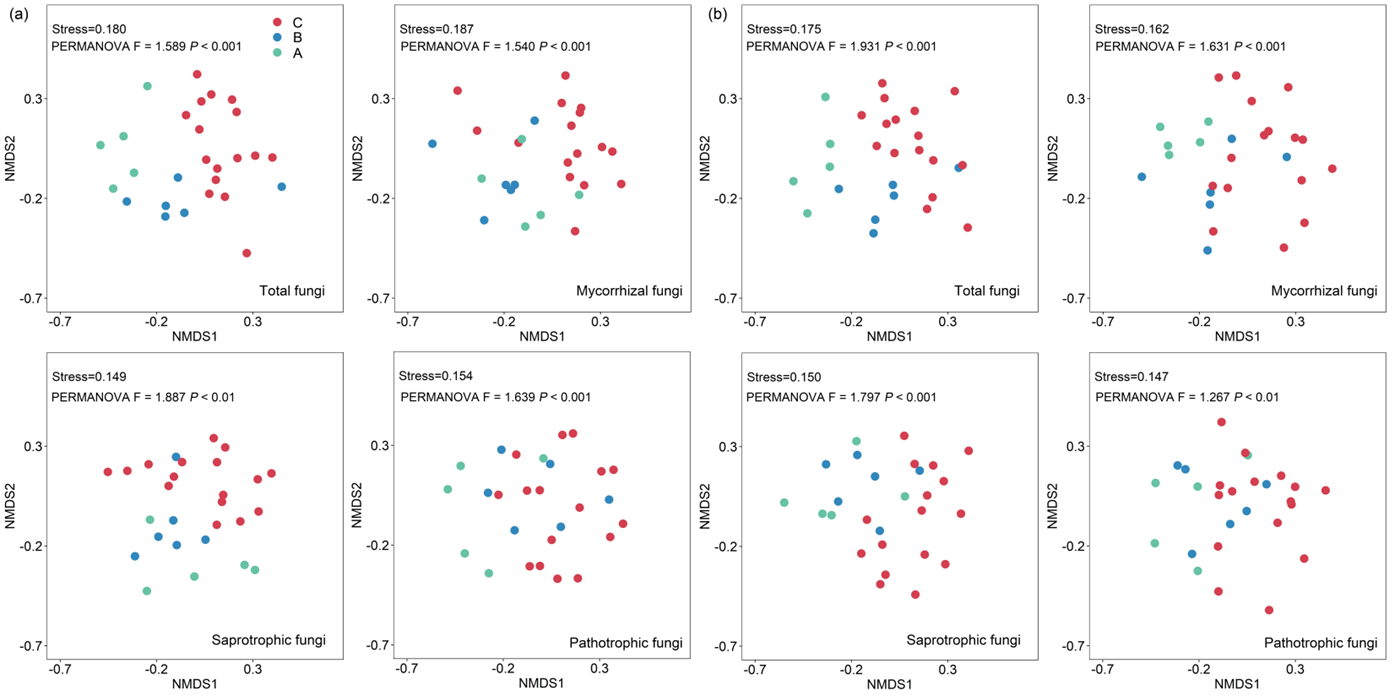


**Figure S3** Fungi community composition for total, mycorrhizal and saprotrophic and pathotrophic fungi in surface (a) and subsurface (b) soil at the site level. The community variation is quantified based on the Bray–Curtis dissimilarity. A, Temperate broadleaf deciduous forest (*Populus euphratica* Woodland); B, Temperate broadleaf deciduous scrub (*Tamarix ramosissima* scrub); C, desert vegetation.

**
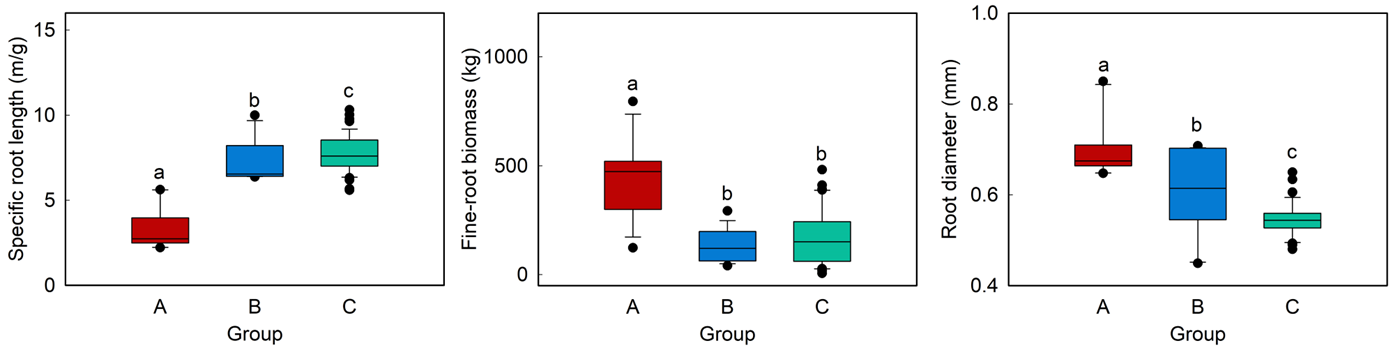
**

**Figure S4** Specific root length, root diameter and fine-root biomass across three vegetation types. The statistical differences among different vegetation types are indicated by different letters at the level of P < 0.05. A, Temperate broadleaf deciduous forest (*Populus euphratica* Woodland); B, Temperate broadleaf deciduous scrub (*Tamarix ramosissima* scrub); C, desert vegetation. Specific root length (SRL) was separated into three categories: low-SRL (A), medium-SRL (B), and high-SRL (C). Fine-root biomass (FRB) were separated into two categories: high-FRB (A), and low-FRB (B and C).


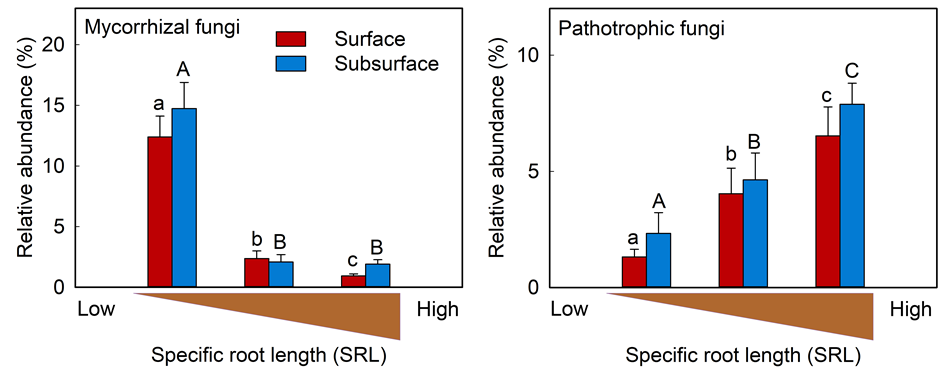


**Figure S5** Variations in relative abundance of mycorrhizal and pathotrophic fungi along specific root length (SRL) gradients in surface and subsurface soil. Boxplots that do not share a letter are significantly different (*P* < 0.05, Wilcoxon rank-sum test).


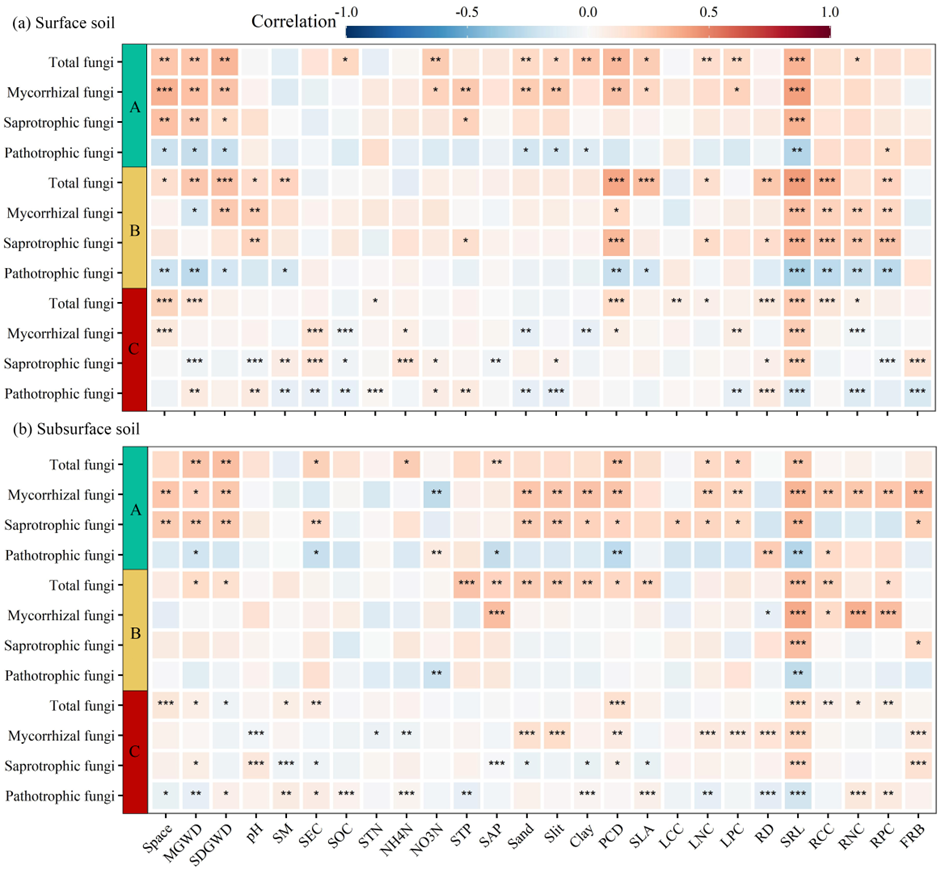


**Figure S6** Mantel test showing relationships between biotic, abiotic factors and β-deviations of total, mycorrhizal, saprotrophic and pathotrophic fungi in surface (a) and subsurface soil (b). See abbreviations in Table S4. A, Temperate broadleaf deciduous forest (*Populus euphratica* Woodland); B, Temperate broadleaf deciduous scrub (*Tamarix ramosissima* scrub); C, desert vegetation.


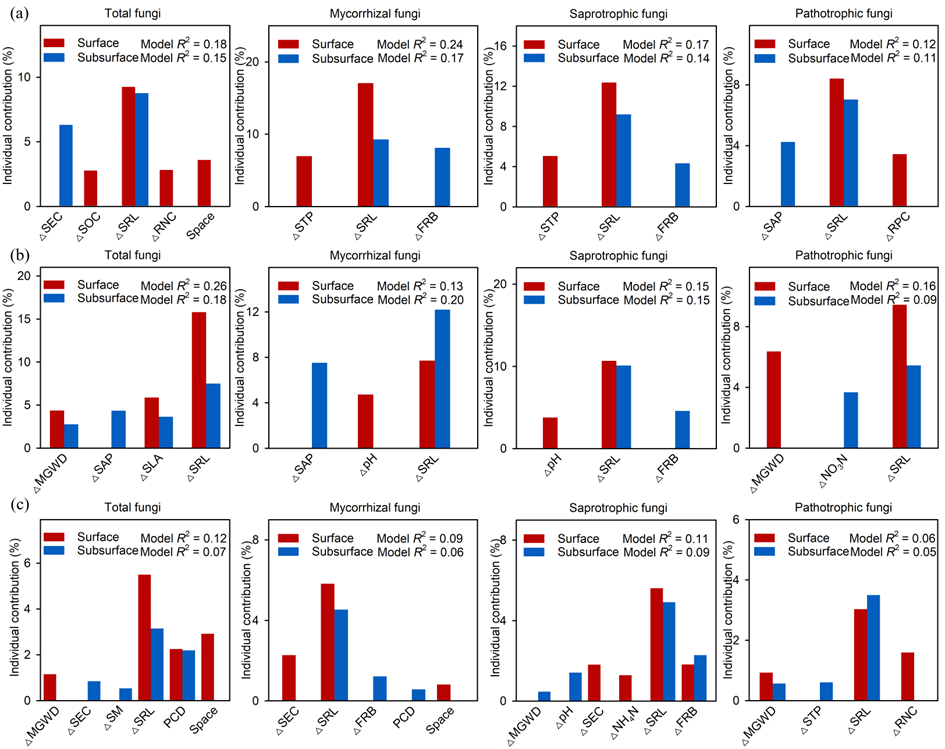


**Figure S7** Results of the multiple regressions on distance matrices (MRM) for β-deviations of total, mycorrhizal, saprotrophic and pathotrophic fungi in surface and subsurface soil in Temperate broadleaf deciduous forest (*Populus euphratica* Woodland) (a), Temperate broadleaf deciduous scrub (*Tamarix ramosissima* scrub) (b) and Desert (c). See abbreviations in Table S4. Space, Spatial distance.
